# Molecular and morphological evidence for *Penstemon luculentus* (Plantaginaceae): a replacement name for *Penstemon fremontii* var. *glabrescens*

**DOI:** 10.3897/phytokeys.63.7952

**Published:** 2016-05-20

**Authors:** Robert L. Johnson, Mikel R. Stevens, Leigh A. Johnson, Matthew D. Robbins, Chris D. Anderson, Nathan J. Ricks, Kevin M. Farley

**Affiliations:** 1 Department of Biology, Brigham Young University, 3115A Monte L. Bean Museum, Provo, Utah 84602 USA; 2 Department of Plant and Wildlife Sciences, Brigham Young University, 5131 Life Sciences Building, Provo, Utah 84602 USA; 3 United States Department of Agriculture, Agricultural Research Service, Forage and Range Research Laboratory, 690 N. 1100 E., Logan, Utah 84322 USA

**Keywords:** Colorado, Rio Blanco, Piceance, White River Shale, Penstemon

## Abstract

*Penstemon luculentus* R.L.Johnson & M.R.Stevens, **nom. nov.** replaces Penstemon fremontii var. glabrescens Dorn & Lichvar. The varietal name *glabrescens* was not elevated because it was already occupied by *Penstemon glabrescens* Pennell, a different species. This new arrangement is supported by molecular and morphological evidence. An analysis of genetic diversity in populations of both varieties of *Penstemon fremontii* Torr. & A. Gray (*glabrescens* and *fremontii*) from the Piceance Basin, Colorado, using SSR (simple sequences repeats) or microsatellites markers, revealed significant genetic differentiation between the two. Penstemon fremontii var. glabrescens was also genetically different from *Penstemon gibbensii* Dorn and Penstemon scariosus var. garrettii (Pennell) N.H. Holmgren. The combination of hirtellous stems, glabrous leaves, non-glandular inflorescence, and long anther hairs distinguish *Penstemon luculentus* from other morphologically similar species.

## Introduction

While investigating *Penstemon scariosus* Pennell (1920) and its varieties, the authors encountered two herbarium specimens from Rio Blanco County, Colorado (BRY81341, BRY81345) that had hirtellous stems, a trait not found in *Penstemon scariosus*. Further investigation led us to determine that the specimens had been misidentified and that they correctly belonged to Penstemon fremontii var. glabrescens Dorn and Lichvar (1990) under existing taxonomic circumscription. Similarly, we encountered several herbarium specimens labeled as *Penstemon gibbensii* Dorn (1982) from Rio Blanco County, Colorado (BRY112313, BRY112314, BRY112315, BRY112316) that also belonged to Penstemon fremontii var. glabrescens. All but one of these specimens was collected prior to the publication of Penstemon fremontii var. glabrescens and they had not been annotated since to reflect this newer taxonomy. The original determinations of these specimens reflect the observed similarity of Penstemon fremontii var. glabrescens to *Penstemon scariosus* and *Penstemon gibbensii*, rather than with *Penstemon fremontii* Torr. & A. Gray in Gray (1862) *sensu stricto*.

Though var. *glabrescens* was recognized at the varietal level within *Penstemon fremontii*, uncertainty as to its placement within this taxon has been expressed. In the most recent treatment of the Colorado Flora: Western Slope, Weber and Wittmann (2012) state, “In our opinion, this variety is not closely related to *Penstemon fremontii* and it might be better placed, as a species, closer to the peripheral *Penstemon scariosus* and *Penstemon gibbensii*.” The similarity of var. *glabrescens* to *Penstemon gibbensii* and *Penstemon scariosus* was also mentioned in its original publication and morphological comparisons made with these taxa (Dorn and Lichvar 1990), although there was no indication with which of the four varieties of *Penstemon scariosus* those comparisons were made.

*Penstemon gibbensii* can be easily distinguished from Penstemon fremontii var. glabrescens by the abundant glandular pubescence present on the inflorescence axis (including sepals and corolla) and distal portions of the stem as compared to the later. The glandular hairs often extend from the distal stem region to mid-stem or below, though becoming less dense proximally. *Penstemon scariosus* only occasionally has glandular hairs (in some varieties) with hairs sparse and never extending onto the proximal portion of the stem. Variety *glabrescens* is most easily distinguished from *Penstemon fremontii* *sensu stricto* by its glabrous leaves and longer-haired anthers versus *Penstemon fremontii* that has hirtellous leaves and shorter anther hairs. Variety *glabrescens* is most easily distinguished from *Penstemon scariosus* by its hirtellous stem, *Penstemon scariosus* having glabrous stems.

In this paper, we re-evaluate some morphological characteristics between *Penstemon fremontii* and Penstemon fremontii var. glabrescens. We also make comparisons against Penstemon scariosus var. garrettii (Pennell 1920) N.H. Holmgren in Cronquist et al. (1984) because it represents a variety of *Penstemon scariosus* that is geographically proximate and of similar floral characteristics. We also compare the genetic structure within and between *Penstemon fremontii* varieties *fremontii* and *glabrescens*, *Penstemon gibbensii*, and Penstemon scariosus var. garrettii from the same region using simple sequence repeat (SSR; i.e., microsatellite markers). These markers are useful in inferring genetic exchange among biological populations (Balloux and Lugon-Moulin 2002). It is our opinion that Penstemon fremontii var. glabrescens is a distinct taxon and should be elevated as a unique species.

## Taxonomic treatment

### Penstemon luculentus

Taxon classificationPlantaeLamialesPlantaginaceae

R.L.Johnson & M.R.Stevens nom. nov.

urn:lsid:ipni.org:names:77154920-1

Penstemon luculentus R.L.Johnson & M.R.Stevens, nom. nov. ≡ Penstemon fremontii Torr. & A. Gray var. glabrescens Dorn & Lichvar, Madroño 37(3): 195–199, f. 1, 2 [map]. 1990. (non Penstemon glabrescens Pennell in Contributions from the United States National Herbarium 20: 375–376. 1920). Type: USA. Colorado: Garfield Co, Douglas Pass, 8000 ft., 7 July 1987, *R. Dorn 4656* (holotype RMS!).

#### Note.

Elevating Penstemon fremontii var. glabrescens to a species using the epithet *glabrescens* was not possible because *Penstemon glabrescens* is already occupied (Pennell 1920).

#### Etymology.

**Figure 1.**
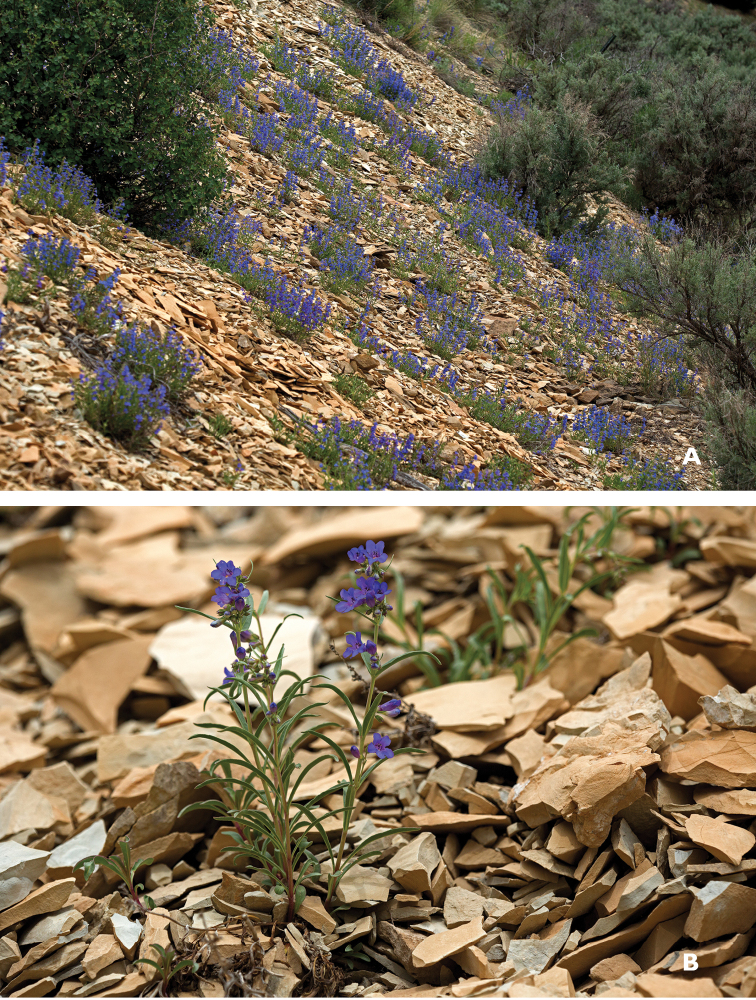
**A** *Penstemon luculentus* in its commonly found native whitish or tan shale habitat **B** An individual *Penstemon luculentus* plant growing in its typical shale habitat.

*Penstemon luculentus* is derived from the Latin “*luculentus*,” meaning brilliant or bright. The name was chosen to reflect the brilliant blue flower color, which is particularly striking in the field contrasting against the whitish or tan shale background typically associated with the species (Fig. 1A, B).

#### Remarks.

**Figure 2.**
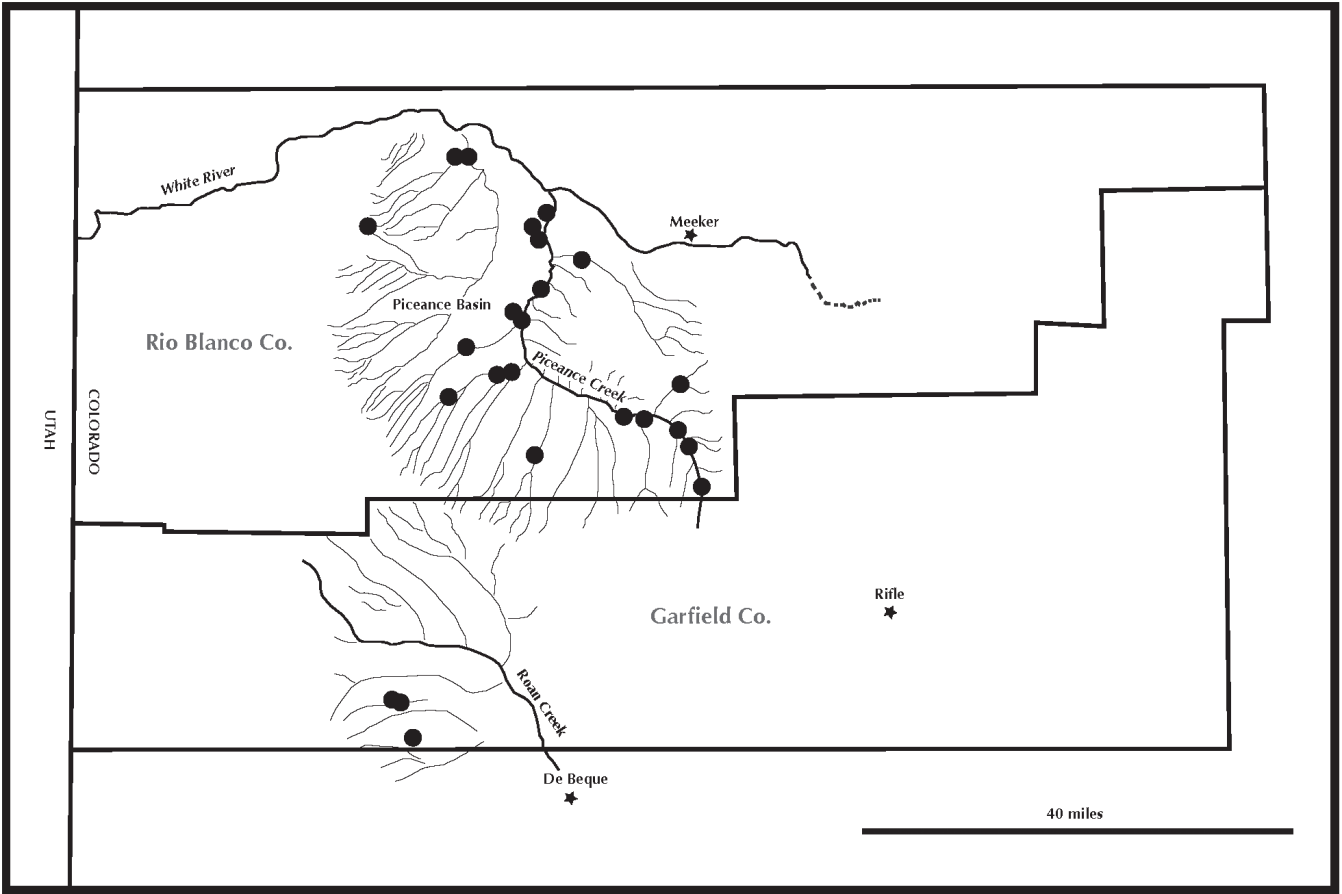
Map showing known distribution of *Penstemon luculentus* in Rio Blanco and Garfield counties Colorado.

*Penstemon luculentus* (≡ Penstemon fremontii var. glabrescens) grows almost exclusively on steep slopes composed of Green River shale or sometimes intermixed with sandstone fragments from overlying strata. It is locally common on road cuts. It occurs primarily within the Piceance drainage with populations occurring abundantly on exposed shale along Piceance Creek and the adjacent tributaries, including the Yellow Creek drainage in Rio Blanco Co., CO. (Fig. 2). It also occurs on shale slopes of the Roan Creek drainage in Garfield Co., CO. The Colorado Natural Heritage Program (CNHP) gives this taxon a global rank of G3G4T2 and a state rank of S2 due to threats from gas and oil drilling throughout its habitat in the Piceance Basin (CNHP 2015). The ranking of G3G4 indicates a status between vulnerable and apparently secure. The rank of S2 specifies a state status of “imperiled – at high risk of extinction due to very restricted range, very few populations (often 20 or fewer), recent and widespread declines, or other factors” (Rondeau et al. 2011). Currently oil and gas drilling have not had a noticeable impact on its populations, but that could change if oil extraction begins to include the mining of oil shale.

## Methods

**Figure 3.**
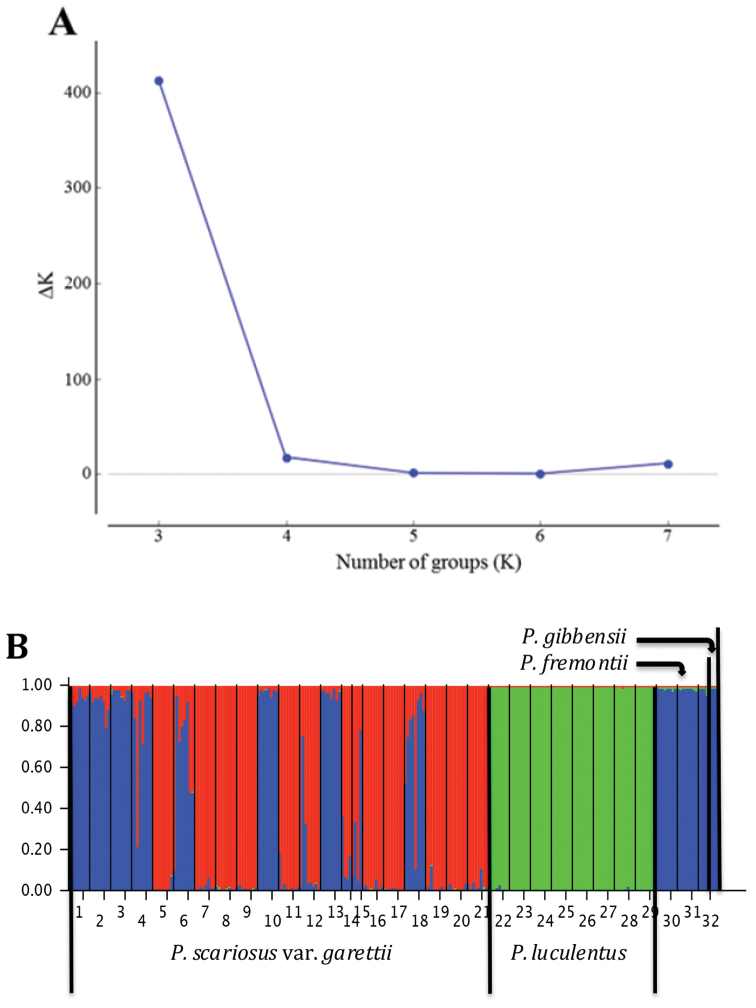
**A** Plot of the second order difference (Δ*K*) of *K* values (2–8) tested in STRUCTURE analysis identifying *K* = 3 as the optimal number of populations based on the accessions of *Penstemon luculentus*, *Penstemon fremontii*, Penstemon scariosus var. garrettii, and *Penstemon gibbensii* tested. As the K values tested were from 2 to 8, the first difference in *K* values (Δ*K*) starts at *K* = 3 **B** Bar plot of inferred ancestry coefficients from STRUCTURE analysis results for with *K* = 3 using 248 samples from 32 accessions. Each number on the x axis represents the accessions ID# in Table 1.

A minimum of one herbarium voucher and four tissue samples were collected at each accession site (Table 1). These samples were collected either during July 2013 or June 2014. DNA extractions were from lyophilized or silica gel dried leaf tissue collected, *in situ* (Table 1), using the method detailed by Todd and Vodkin (1996). We used the same PCR parameters and ten of the fluorescently labeled primers (Table 2.) reported by Anderson et al. (2016) to run each DNA sample. Furthermore, we followed their protocol using Geneious 8.0.5 (Kearse et al. 2012) to score the output generated from the ABI 3730xl (Applied Biosystems, Carlsbad, CA, USA) at Brigham Young University’s DNA Sequencing Center (Provo, UT, USA) for the population genetic structure study (Fig. 3A, B).

**Table 1.** Identification number (ID#) and geographic origin of the 32 accessions of *Penstemon* included in this study. Vouchers for each accession were deposited in the Stanley L. Welsh Herbarium (BRY), Brigham Young University Provo, Utah, USA.

ID#	Taxon	N	Accession location	Latitude	Longitude	Voucher no.
1	Penstemon scariosus var. garrettii	8	North of Little Mountain Peak, Sweetwater Co., WY	41°10'58.4"N	109°16'51.7"W	BRY121014
2	Penstemon scariosus var. garrettii	8	Goslin Mountain, Daggett Co., UT	40°56'44.5"N	109°15'35.1"W	BRY121028
3	Penstemon scariosus var. garrettii	8	North of Lone Tree, Uinta Co., WY	41°05'10.1"N	110°11'19.3"W	BRY121027
4	Penstemon scariosus var. garrettii	8	Oilfield Reservoir area, Moffat Co., CO	40°39'14.9"N	109°00'24.7"W	BRY119254
5	Penstemon scariosus var. garrettii	8	Price Canyon, Utah Co., UT	39°49'43.2"N	110°57'28.0"W	BRY117079
6	Penstemon scariosus var. garrettii	8	South of Manila, Daggett, Co., UT	40°52'56.1"N	109°41 ‘33.5"W	BRY117080
7	Penstemon scariosus var. garrettii	8	East of Fruitland, Duchesne Co., UT	40°12'15.7"N	110°47'57.1"W	BRY133591
8	Penstemon scariosus var. garrettii	8	Midway, Wasatch Co., UT	40°32'03.2"N	111°28'57.7"W	BRY117064
9	Penstemon scariosus var. garrettii	8	Northeast of Birdseye, Utah, Co., UT	39°55'38.0"N	111°32'37.0"W	BRY124358
10	Penstemon scariosus var. garrettii	8	Argyle Canyon, Duchesne Co., UT	39°53'44.3"N	110°38'18.7"W	BRY121021
11	Penstemon scariosus var. garrettii	8	Northwest of Whiterocks, Duchesne Co., UT	40°35'45.1"N	110°06'06.1"W	BRY113493
12	Penstemon scariosus var. garrettii	8	Pine Mountain, Sweetwater Co., WY	41°03'42.5"N	108°57'45.0"W	BRY121020
13	Penstemon scariosus var. garrettii	4	along HWY 191 North of Vernal, Uintah Co., UT	40°39'41.4"N	109°28'50.1"W	BRY121013
14	Penstemon scariosus var. garrettii	4	along HWY 191 North of Vernal, Uintah Co., UT	40°42'41.5"N	109°29'38.0"W	BRY121026
15	Penstemon scariosus var. garrettii	8	Sowers Canyon, Duchesne Co., UT	39°55'21.5"N	110°35'13.7"W	BRY119259
16	Penstemon scariosus var. garrettii	8	Yellowstone Creek Drainage, Duchesne Co., UT	40°33'00.5"N	110°19'16.4"W	BRY119253
17	Penstemon scariosus var. garrettii	8	Head of Warner Draw, Uintah Co., UT	40°44'52.9"N	109°13'41.6"W	BRY119256
18	Penstemon scariosus var. garrettii	8	Red Cloud Loop, Uintah Co., UT	40°37'28.7"N	109°45'38.8"W	BRY119261
19	Penstemon scariosus var. garrettii	8	Cat Peak, Utah Co., UT	39°53'56.8"N	110°57'34.0"W	BRY109209
20	Penstemon scariosus var. garrettii	8	Willow Creek Guard Station area, Wasatch Co., UT	40°02'36.2"N	111°08'59.2"W	BRY119260
21	*Penstemon luculentus*	8	Piceance Canyon, Rio Blanco Co., CO	39°45'42.4"N	108°00'46.4"W	BRY126454
22	*Penstemon luculentus*	8	Piceance Canyon, Rio Blanco Co., CO	39°48'03.2"N	108°07'28.9"W	BRY130985
23	*Penstemon luculentus*	8	Piceance Canyon, Rio Blanco Co., CO	39°51'31.5"N	108°18'47.5"W	BRY130983
24	*Penstemon luculentus*	8	Piceance Canyon, Rio Blanco Co., CO	39°49'36.4"N	108°25'06.8"W	BRY130982
25	*Penstemon luculentus*	8	Piceance Canyon, Rio Blanco Co., CO	39°53'40.1"N	108°23'29.7"W	BRY130981
26	*Penstemon luculentus*	8	Piceance Canyon, Rio Blanco Co., CO	39°55'40.1"N	108°17'36.4"W	BRY130980
27	*Penstemon luculentus*	8	Piceance Canyon, Rio Blanco Co., CO	40°00'26.2"N	108°11'33.8"W	BRY130979
28	*Penstemon luculentus*	8	Piceance Canyon, Rio Blanco Co., CO	40°03'51.4"N	108°15'06.7"W	BRY126453
29	*Penstemon fremontii*	8	Near Meeker, Rio Blanco Co., CO	39°58'59.1"N	107°58'02.6"W	BRY121022
30	*Penstemon fremontii*	8	Piceance Canyon, Rio Blanco Co., CO	39°48'19.7"N	108°05'16.1"W	BRY104606
31	*Penstemon fremontii*	8	Piceance Canyon, Rio Blanco Co., CO	39°53'27.8"N	108°10'47.9"W	BRY104599
32	*Penstemon gibbensii*	8	Browns Park, Daggett Co., UT	40°50'49.1"N	109°02'59.3"W	BRY28472

Note: N = number of tissue samples for each accession.

**Table 2.** The ten SSR markers used in this study with associated variability of each marker relative to each taxon and across taxa.

	Taxon												Allele totals	
	*Penstemon fremontii* (N=24)			*Penstemon luculentus* (N=64)			*Penstemon gibbensii* (N=8)			Penstemon scariosus var. garrettii (N=152)				
**Locus**	A	A_U_	Size range (bp)	A	A_U_	Size range (bp)	A	A_U_	Size range (bp)	A	A_U_	Size range (bp)	A_C_	A_T_
**Pen04**	17	1	216-252	24	18	215-254	3	0	218-248	20	2	212-252	17	38
**Pen23**	11	0	158-184	14	0	154-190	6	0	160-174	23	8	150-195	15	23
**PS014**	7	1	211-236	12	2	214-239	2	1	219-221	16	4	209-242	12	20
**PS016**	13	0	150-170	20	1	149-173	6	1	161-168	30	11	136-189	21	34
**PS048**	1**^†^**	0	225	2	0	213-225	3	0	225-233	10	6	213-245	4	10
**PS077**	5	0	118-139	6	1	123-145	3	1	134-150	9	2	118-145	7	11
**PS079**	14	7	160-201	14	3	139-201	3	1	135-148	14	3	133-175	13	27
**PS080**	7	1	212-228	19	4	213-238	3	0	218-223	23	10	196-242	15	30
**PS082**	14	2	164-219	19	3	192-217	3	0	205-212	21	5	168-224	19	29
**PS084**	5	0	118-138	12	8	117-143	2	0	118-128	7	1	118-148	6	15

Note: N = number of samples for each taxon, A = number of alleles observed in a given taxon, A _U_ = number of alleles unique to a given taxon, A _C_ = number of alleles shared between two or more taxa, A _T_ = total number of alleles identified in this study for a given marker. ^†^Locus was monomorphic.

**Figure 4.**
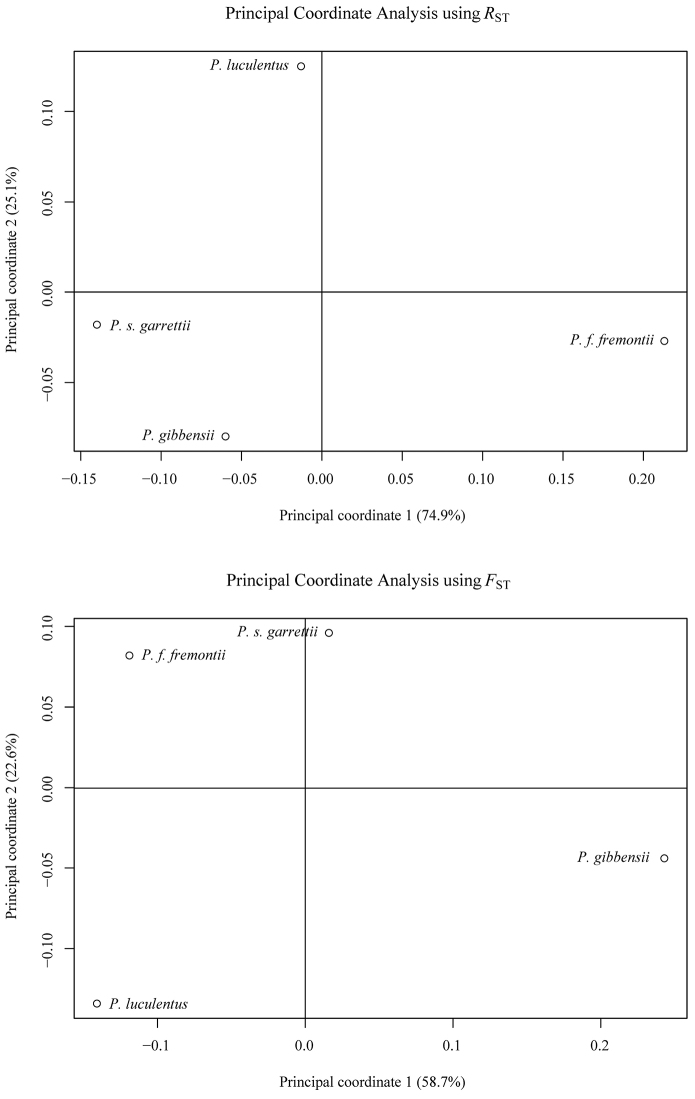
Plots of eigenvectors of the first two coordinates of principal coordinate analysis based on pairwise *R*_ST_ (top graph) or *F*_ST_ (bottom graph) values computed from genotypes of ten SSR markers on all taxa. Numbers in parentheses on each axis indicate the percent variation explained by each coordinate.

To understand the population genetic structure of the accessions we sampled (Table 1), we used STRUCTURE 2.3 (Falush et al. 2003; Pritchard et al. 2000). The optimal number of genetically distinct clusters or groups (*K*) was determined by testing *K* values from 2 to 8 (1 was not tested as multiple clusters were expected) and plotting the second order difference (Δ*K*) between each *K* value (Fig. 3A) according to Evano et al. (2005). Analyses consisted of 10 iterations using a burnin period of 50,000 reps with 1,000,000 MCMC reps following burnin, admixture assumed, and sampling locations used as priors. Genetic diversity was partitioned using an analysis of molecular variance (AMOVA) implemented in GenAlEx 6.501 (Peakall and Smouse 2012) to compute pairwise *F*_ST_ and *R*_ST_ values between taxa (Table 3). The AMOVA was implemented using 999 permutations to calculate P-values for each *F*_ST_ or *R*_ST_ value. Both pair-wise matrices were then used in GenAlEx to conduct principal coordinate analyses (PCoA) to visualize the differences between taxa (Fig. 4).

**Table 3.** *R*_ST_ and *F*_ST_ values (bottom diagonals) with accompanying P-values (top diagonals) for the pairwise comparisons of *Penstemon luculentus*, *Penstemon fremontii*, Penstemon scariosus var. garrettii, and *Penstemon gibbensii*.

Pairwise population *R*_ST_ values				
	Taxon			
Taxon	Penstemon scariosus var. garrettii	*Penstemon luculentus*	*Penstemon fremontii*	*Penstemon gibbensii*
**Penstemon scariosus var. garrettii**	0.000	0.001	0.001	0.154
***Penstemon luculentus***	0.060	0.000	0.001	0.031
***Penstemon fremontii***	0.215	0.127	0.000	0.026
***Penstemon gibbensii***	0.013	0.076	0.132	0.000
**Pairwise population *F*_ST_ values**				
**Penstemon scariosus var. garrettii**	0.000	0.001	0.001	0.001
***Penstemon luculentus***	0.148	0.000	0.001	0.001
***Penstemon fremontii***	0.124	0.117	0.000	0.001
***Penstemon gibbensii***	0.170	0.279	0.262	0.000

**Figure 5.**
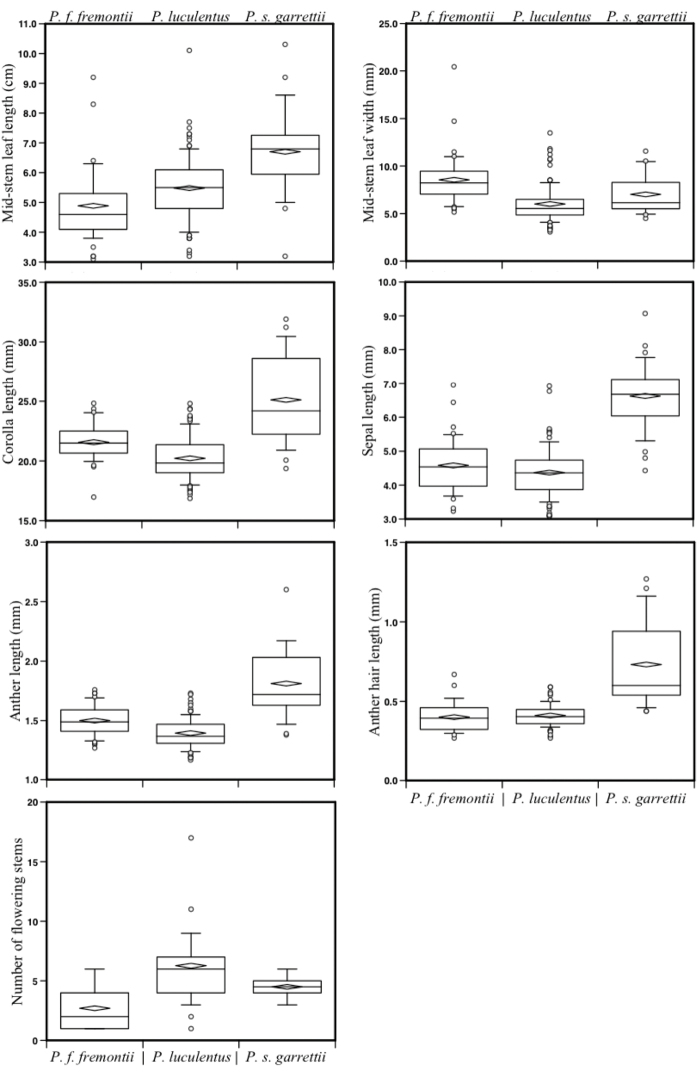
Box percentile plots showing variations among plant characteristics between *Penstemon fremontii*, *Penstemon luculentus*, and Penstemon scariosus var. garrettii. Boxes delimit the 75th and 25th percentiles. The whiskers delimit the 10th and 90th percentile with outliers shown as circles outside the whiskers. The horizontal bar shows the 50th percentile and the horizontal triangle is the mean.

We made morphological comparisons, using field-collected plants and herbarium specimens obtained from the Stanley L. Welsh Herbarium (BRY) and Rocky Mountain Herbarium (RMS). We took multiple measurements from 38 herbarium sheets of Penstemon fremontii var. glabrescens (≡ *Penstemon luculentus*) including the holotype and four paratypes, and 20 sheets each of *Penstemon fremontii* *sensu stricto* and Penstemon scariosus var. garrettii. Sheet selection was based on the specimen completeness (i.e. only entire plant(s), not partial plants) and the specimen’s pressed condition. Accurate floral measurements required corollas to have dried completely pressed without shrinkage. Sheets of *Penstemon fremontii* and Penstemon scariosus var. garrettii were selected from the same or adjacent counties to Rio Blanco Co. in Utah and Colorado. Small measurements were taken from digital images with an Olympus SZX-16 dissecting microscope and processed using CellSens Standard 1.8 imaging platform (Olympus Corporation). Because of size similarities between measured plant characteristics, data were plotted as box percentile plots (Fig. 5) with the boxes delimiting the 75th and 25th percentiles and whiskers delimiting the 10th and 90th percentile. Outliers were shown as circles outside the whiskers. We did not have enough material to include *Penstemon gibbensii*.

## Results and discussion

We first analyzed the SSR data, between, and within specimens of *Penstemon luculentus*, *Penstemon fremontii*, Penstemon scariosus var. garrettii, and *Penstemon gibbensii* (Table 1) using STRUCTURE. The results revealed that the best *K* value for these taxa was *K* = 3 and at that *K* value, *Penstemon luculentus* distinctly differed in population genetic composition from any of the other morphologically similar species (Fig. 3A, B). All eight sites (64 specimens) of *Penstemon luculentus* sampled across the plant’s range were similar in genetic composition. Varying levels of admixture were detected among sites of Penstemon scariosus var. garrettii. Some sites genetically resemble *Penstemon gibbensii* and *Penstemon fremontii* with inferred ancestry coefficients of all specimens of 0.9 or greater for the *Penstemon gibbensii* and *Penstemon fremontii* group (blue in Fig. 3B). However, some sites were genetically distinct from all other species with inferred ancestry coefficients of all specimens of 0.9 or greater for their own Penstemon scariosus var. garrettii group (red in Fig. 3B). Still other sites contained specimens that varied in their relatedness to either of these two groups. *Penstemon fremontii* showed greater genetic similarity to *Penstemon gibbensii* and Penstemon scariosus var. garrettii than with *Penstemon luculentus*. This genetic similarity may be due to several factors, such as a possible common ancestor or historical recombination between species. The elucidation of the factors involved in creating these genetic relationships is beyond the scope of this work and requires further research.

To gain an improved understanding of the relationships between *Penstemon luculentus*, *Penstemon fremontii*, Penstemon scariosus var. garrettii, and *Penstemon gibbensii*, we analyzed the SSR allele results using AMOVA (analysis of molecular variance). The analysis revealed that, based on *F*_ST_, molecular variance was partitioned as 15% among taxa, 26% among individuals across taxa, and 59% within individuals of the same taxa, with an overall *F*_ST_ of 0.149 (P-value = 0.001). For the AMOVA analysis based on *R*_ST_, molecular variance was partitioned as 11% among taxa, 78% among individuals, and 11% within individuals, with an *R*_ST_ value of 0.106 (P-value = 0.002). All pair-wise *F*_ST_ and *R*_ST_ values were statistically significant except for the *R*_ST_ value of *Penstemon gibbensii* and Penstemon scariosus var. garrettii (Table 3). Analysis with both *F*_ST_ and *R*_ST_ indicated that *Penstemon luculentus* has a unique genetic composition as compared to the other taxa which is illustrated in the graphs of the first two coordinates of the PCoA analyses (Fig. 4). These results support the validity of *Penstemon luculentus* being recognized as a unique species distinct from *Penstemon fremontii* *sensu stricto.* The *F*_ST_ analysis suggests that Penstemon scariosus var. garrettii and *Penstemon fremontii* are more closely related than either are to *Penstemon gibbensii*, while the *R*_ST_ analysis suggests that Penstemon scariosus var. garrettii and *Penstemon gibbensii* are more similar. This discrepancy suggests that microsatellite mutations, which are modeled in the stepwise mutation model of *R*_ST_ (reviewed by Balloux and Lugon-Moulin 2002), contribute to genetic differentiation among the taxa examined. The determination of the mutation rates of each SSR locus is beyond the scope of this study, but should be considered in future analyses with these loci.

Morphological comparisons revealed overlap in the size of many plant characters between *Penstemon luculentus*, *Penstemon fremontii*, and Penstemon scariosus var. garrettii. Even though there was overlap in the range of measured characteristics, the means do reveal segregating features (Fig. 5). Overall, *Penstemon luculentus* had more flower stems, a smaller caulescent leaf width, a smaller corolla, and a smaller anther cell length but was found to be intermediate in caulescent leaf length. While *Penstemon luculentus* was similar to *Penstemon fremontii* in sepal and anther hair length, these characters were much shorter than those found in Penstemon scariosus var. garrettii.

## Conclusion

While *Penstemon luculentus* has similar morphologically characteristics to *Penstemon fremontii*, and Penstemon scariosus var. garrettii, there are distinctions that can reliably segregate these taxa. Distinguishing characteristics are more apparent when comparing these taxa *in situ*. The combination of hirtellous stems, glabrous leaves, non-glandular inflorescence, and long anther hairs can be used to segregate *Penstemon luculentus* from other related taxa. Differences in other morphological characters are subtler, largely observed as differences in the means of their measurements, and are not reliably diagnostic.

Molecular evidence suggests that *Penstemon luculentus* is distinct from *Penstemon fremontii* *sensu stricto*. It is also distinct from Penstemon scariosus var. garrettii and *Penstemon gibbensii*. While *Penstemon luculentus* is not sympatric with Penstemon scariosus var. garrettii, it is well within the geographic range of *Penstemon fremontii*. We observed *Penstemon luculentus* and *Penstemon fremontii*, growing naturally, within 100 m of each other with no apparent hybridization between them. Although we did not observe the two taxa growing interlaced, it is possible that they could co-occur in some areas of the Piceance Basin. Despite both *Penstemon luculentus* and *Penstemon fremontii* commonly occurring in the Piceance Basin, there was no morphological evidence that these taxa are exchanging alleles even though they are blooming simultaneously. The results of our study of both the SSR and morphometric data indicate that *Penstemon luculentus* should be elevated to species status.

### Taxonomic key

*Penstemon luculentus* can be segregated from *Penstemon fremontii*, *Penstemon scariosus*, and *Penstemon gibbensii* using the following key. We don’t attempt to segregate the different varieties of *Penstemon scariosus* in this key but recognize where they would segregate from *Penstemon luculentus*. The taxonomic status of the varieties of *Penstemon scariosus* is currently being investigated.

**Table:** 

1	Stems hirtellous, eglandular	**2**
–	Stems glabrous or with hairs glandular and only occurring distally or on inflorescence axis	**3**
2	At least some leaf blade surfaces hirtellous, basal leaves spatulate to broadly oblanceolate, usually present at anthesis	***Penstemon fremontii***
–	Leaf blades glabrous or with scabrous hairs restricted to leaf margins, basal leaves linear to lanceolate when present, usually absent at anthesis	***Penstemon luculentus***
3	Distal portion of stem and inflorescence axis with glandular hairs	**4**
–	Distal portion of stems and inflorescence axis glabrous	**Penstemon scariosus var. scariosus , Penstemon scariosus var. garrettii**
4	Sepals < 5mm, glandular hairs abundant	***Penstemon gibbensii***
–	Sepals 5–6+ mm, glandular hairs sparse	**Penstemon scariosus var. albifluvis , Penstemon scariosus var. cyanomontanus , occasionally Penstemon scariosus var. garrettii**

## Supplementary Material

XML Treatment for Penstemon luculentus
